# Contact Lenses Delivering Nitric Oxide under Daylight for Reduction of Bacterial Contamination

**DOI:** 10.3390/ijms20153735

**Published:** 2019-07-31

**Authors:** Mimimorena Seggio, Antonia Nostro, Giovanna Ginestra, Fabiana Quaglia, Salvatore Sortino

**Affiliations:** 1 Laboratory of Photochemistry, Department of Drug Sciences, University of Catania, 95124 Catania, Italy; 2 Department of Chemical, Biological, Pharmaceutical and Environmental Sciences, University of Messina, 98122 Messina, Italy; 3 Drug Delivery Laboratory, Department of Pharmacy, University of Napoli Federico II, 80138 Napoli, Italy

**Keywords:** photorelease, nitric oxide, fluorescence, contact lens, antibacterial

## Abstract

Ocular infection due to microbial contamination is one of the main risks associated with the wearing of contact lens, which demands novel straightforward strategies to find reliable solutions. This contribution reports the preparation, characterization and biological evaluation of soft contact lenses (CL) releasing nitric oxide (NO), as an unconventional antibacterial agent, under daylight exposure. A tailored NO photodonor (NOPD) was embedded into commercial CL leading to doped CL with an excellent optical transparency (transmittance = 100%) at λ ≥ 450 nm. The NOPD results homogeneously distributed in the CL matrix where it fully preserves the photobehavior exhibited in solution. In particular, NO release from the CL and its diffusion in the supernatant physiological solution is observed upon visible light illumination. The presence of a blue fluorescent reporting functionality into the molecular skeleton of the NOPD, which activates concomitantly to the NO photorelease, allows the easy monitoring of the NO delivery in real-time and confirms that the doped CL work under daylight exposure. The NO photoreleasing CL are well-tolerated in both dark and light conditions by corneal cells while being able to induce good growth inhibition of *Staphylococcus aureus* under visible light irradiation. These results may pave the way to further engineering of the CL with NOPD as innovative ocular devices activatable by sunlight.

## 1. Introduction

Contact lenses (CL), commonly employed to correct refractive errors, have been recently proposed as a drug delivery platform allowing constant and sustained drug levels in the eye [1,2,3]. After loading with therapeutics, CL can be used to treat specific ocular pathologies and persistent epithelial defects of the cornea, as well as to aid in healing post-surgical corneal wounds. In fact, stagnation in the post-lens tear fluid promotes drug accumulation on the cornea surface and prompt penetration of delivered drug toward the aqueous humor [1,2,3].

Whilst CL wearing is generally safe, it can lead to ocular inflammation and infection due to microbial contamination. Colonization by Gram-positive (such as *Staphylococcus aureus*) or Gram-negative (such as *Pseudomonas aeruginosa*) bacteria increases the risk of initiating ocular inflammation and even infection [4,5]. Due to adhesion of microbes to CL and their prolonged contact on the cornea, risk of infection is high [6]. Furthermore, impaired corneal barrier functions due to underlying pathologies, hypoxic environment on the cornea and reduced exchange of tear fluid, are all factors dampening the response to infection [7]. Thus, intense effort is nowadays devoted to find effective strategies to solve this issue.

Over the past 20 years, several groups have been developing antimicrobial CL, CL cases, or ways of detecting microbial contamination so that users can dispose of lenses or cases when they become contaminated. Lakshminarayanan et al. recently published an excellent review paper regarding this [8]. Silver-ion impregnated contact lens cases, or colloidal silver-impregnated CL have been developed so far [9], although there is no clinical evidence of their ability to reduce bacterial burden. Other bioactive compounds as antimicrobial peptides [10], quorum-sensing inhibitors [11], and non-steroidal anti-inflammatory drugs [12] have been incorporated in CL to prevent microbial biofilm production.

Strategies to generate ephemeral cytotoxic species to kill bacteria while wearing CL could represent a valid alternative to conventional antibiotic therapies. In this context, the use of nitric oxide (NO)-releasing agents is particularly promising. In fact, besides playing a pleiotropic role in the bioregulation of important physiological processes [13], this small sized and uncharged free radical offers a combination of ideal properties for antibacterial applications such as (i) multitarget action with broad-spectrum antibacterial activity [14], (ii) absence of multidrug resistance issues [15], and (iii) short half-life (<1 s) and short diffusion in the cellular environment over short distances (<200 µm), [13] confining the region of action and consequently avoiding systemic toxicity issues. Therefore, the development of CL integrating NO donors stimulated by light inputs, namely NO photodonors (NOPD) [16,17,18] is particularly appealing. In contrast to application in other pathologies such as cancer, the use of phototherapeutic systems such as NOPD in the ocular field does not meet the drawbacks due to poor visible light penetration and, as a consequence, does not necessarily require the development of chromogenic units strictly activatable in the phototherapeutic window. Therefore, the development of CL capable of delivering NO under the input of daylight represents an appealing and surprisingly underexplored way to design smart CL with long-lasting antimicrobial activity. Furthermore, since NO has also a well-recognized role in promoting corneal epithelial wound healing in cell and animal models [19] the concept of sustained NO release from bandage contact lenses could enlarge the arsenal of therapeutic tools available so far. In fact, photocontrolled NO delivery results in a fine tuning of NO levels on corneal epithelium, which is critical since only low concentrations significantly enhance corneal cell viability and promote wound healing [19].

Stimulated by our ongoing interest in the design and fabrication of NO photoreleasing constructs [16,20,21,22,23,24], we report herein the first example of CL incorporating a tailored NOPD generating NO with a fluorescent reporting function under daylight exposure, its tolerability by corneal cells and photo-antibacterial activity against *S. aureus*.

## 2. Results and Discussion

**Scheme 1:**
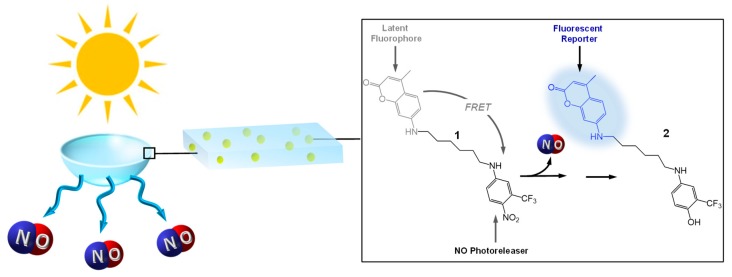
Schematic for the nitric oxide (NO)-photoreleasing contact lenses (CL) loaded with the NO photodonor (NOPD) **1** and its working principle.

One of the main pre-requisites for photoactivatable CL is their effective absorption of daylight below 450 nm without affecting the normal vision. In searching for a suitable NOPD fulfilling the above requisite and to easily incorporate the NOPD into CL without sophisticated synthetic procedures, we decided to use the molecular hybrid **1** (Scheme 1). This compound, recently developed in our group [25], consists of a nitroaniline-based NO photoreleaser unit [26,27] linked, through an alkyl spacer, to coumarin 120, which acts as a fluorescent reporter for the NO release. In fact, the typical blue fluorescence of the coumarin is significantly quenched in the conjugate **1** by a Förster resonance energy transfer (FRET) mechanism. Excitation with visible light triggers NO photorelease from the nitroaniline moiety leading to the phenol derivative **2** as a stable byproduct. In contrast to compound **1**, in the photoproduct **2** FRET is not allowed and, as a consequence, the coumarin fluorescence emission is fully restored [25]. This makes the NO release process easily monitored by the blue fluorescence emission of the reporter, which acts exactly as an optical counter of the bioactive species and, in the case of CL, can provide not only an immediate read-out that they are working properly but also an indication of the exhaustion of the NO reservoir.

**Figure 1:**
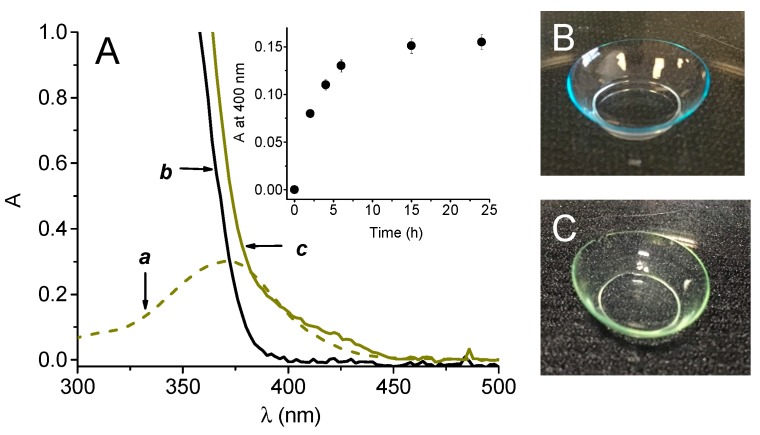
(**A**) Absorption spectra of an ethanol solution of **1** (**a**) and the CL before (**b**) and after (**c**) loading with **1**. (**B**) and (**C**) show the actual images of the CL before and after loading with **1**, respectively.

According with the pre-requisites illustrated above, compound **1** absorbs significantly above 380 nm ((**a**) in Figure 1A), where the typical light transmission of CL is ca. 100% ((**b**) in Figure 1A) but not above 450 nm. These spectral features ensure, in principle, a satisfactory absorption of daylight and a transmittance of 100% in the almost entire visible range without hampering the normal vision. Compound **1** is water insoluble but can be easily loaded into CL by an ethanol solution (see experimental). Spectrum **c** in Figure 1A shows the effective entrapment of **1** in the polymeric matrices of the CL and the absence of any scattering above 450 nm confirm its excellent solubilization without any signs of precipitation within the CL. As shown in the inset of Figure 1A, the loading was complete already after about 6 h. The loading procedure was highly reproducible and gave an average of the amount of **1** in the CL of 3.4 µg/CL (ca. 0.01% by wt, see experimental). The actual images of the CL taken before and after the loading witness that the CL turns from colorless to a pale yellow ensuring an optical transparency of 100% at wavelengths >450 nm. The loaded CL did not show any significant leaching of compound **1** after immersion in artificial tears solution for 24 h.

**Figure 2:**
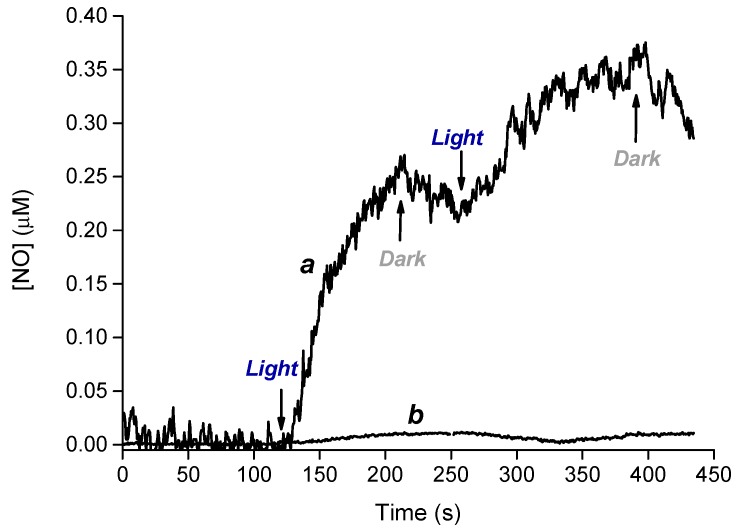
NO release profile observed upon irradiation at 405 nm (100 mW) of CL loaded (**a**) and unloaded (**b**) with compound **1** and immersed in 3 mL of artificial tears solution.

Preservation of the photochemical properties of photoresponsive molecules after incorporation in a host environment is not obvious. In fact, it is well-known that polarity, steric constrains and specific interactions can often lead to dramatic changes in the nature, efficiency or both of the main photochemical processes observed for the free molecules [28,29]. Therefore, we firstly evaluated the NO release upon visible light excitation of the loaded CL. The best method at this regard is the direct detection of this species by amperometric detection using an ultrasensitive NO electrode. To this end, we used a 405 nm continuum laser with a very small diameter of the laser spot (ca. 1.5 mm). This allows the light path to be positioned outside the electrode position avoiding NO signal artifacts due to photoelectric interference on the NO electrode. Direct NO detection from CL was carried out immobilizing the lens to one of the walls of the quartz cell and submerging in 3 mL of artificial tears solution (see experimental). Figure 2 shows that generation of NO from the CL and its diffusion into solution occurs exclusively upon light excitation; it stops in the dark and restarts again as the light is turned on.

**Figure 3:**
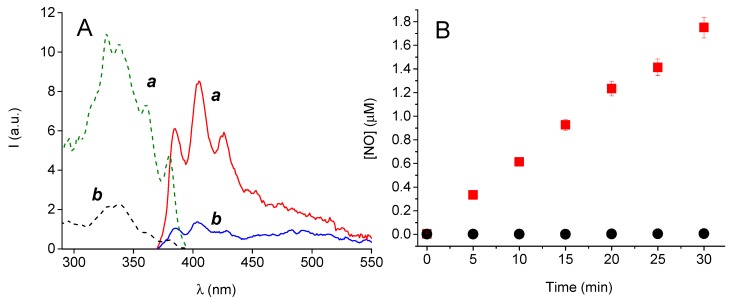
(**A**) Representative fluorescence emission (solid, λ_exc_ = 360 nm) and excitation (dotted, λ_em_ = 405 nm) spectra obtained after fluorimetric assay of CL loaded with **1**, immersed in 3 mL of artificial tears solution, and exposed to daylight (**a**) or kept in the dark (**b**). (**B**) NO concentration, determined by the fluorimetric assay, observed after exposure of the CL loaded with **1** to daylight for different times (■) or kept in the dark (●).

The capability of the CL to release NO under daylight exposure was tested by an indirect method using the 2,3-diaminonaphthalene (DAN), which is one of the most sensitive and selective fluorescence-based methods for NO detection as nitrite (see experimental). Loaded CL were immersed in 3 mL of artificial tears solution and either exposed to daylight during a sunny day between 1:00 and 3:00 pm or kept in the dark. Figure 3A shows that the appearance of the typical fluorescence emission and excitation spectrum of the highly fluorescent 2,3-diaminonaphthotriazole (DANT) formed after reaction of DAN with nitrite is observed only for the CL exposed to light. Experiments carried out at different irradiation times showed an excellent linear correlation of the NO photoreleased with the time exposure (Figure 3B).

**Figure 4:**
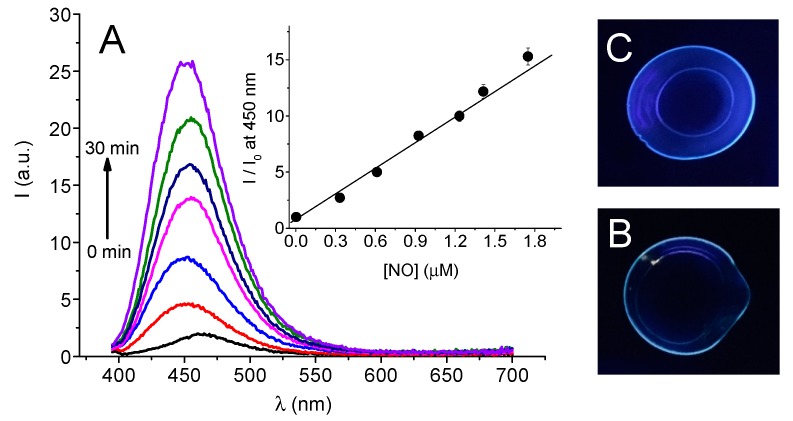
(**A**) Fluorescence emission (λ_exc_ = 380 nm) spectral changes observed for CL loaded with **1** exposed to daylight at regular intervals of five min. The inset shows the correlation of the fluorescence increase and the concentration of NO released from the CL (I and I_0_ represent the fluorescence intensities after and before the irradiation, respectively). (**B**) and (**C**) show the actual fluorescence images of the CL (diameter = 14 mm) observed upon 350 nm of light excitation before and after 30 min exposition of the CL to daylight, respectively.

Thanks to the presence of the coumarin fluorescent reporter in the structure of **1**, the NO release process triggered by sunlight can be also followed by monitoring directly the fluorescence of the loaded CL. Figure 4A clearly shows that, according to the FRET process occurring in **1** (see Scheme 1), the non-irradiated CL show a negligible fluorescence which, in contrast, increases dramatically upon exposure of the CL to daylight. This finding confirms that the formation of the highly fluorescent product **2** concomitantly to the NO release occurs also within the CL polymeric network. As illustrated in the inset of Figure 4A, we found an excellent linear correlation between the concentration of NO photogenerated and the increase of the fluorescence intensity of the optical reporter. Note that, the amount of NO released after 30 min exposure represents about 0.2% of the total NO reservoir available in the CL. As a consequence, even by considering a constant sunlight flux for the whole day, the loaded NOPD results in more than enough to ensure NO photorelease during one full day of light exposure. Interestingly, the process can be also followed at the naked eye. Figure 4B,C show the actual images of CL (350 nm light excitation) before and after 30 min of daylight exposure where it is possible to note the unambiguous increase of the blue fluorescence of the optical reporter in the case of the irradiated sample. Such a fluorescence increase of the CL after irradiation confirms well that the photoproduct **2** remains entrapped therein. This was further supported by the absence of any detectable fluorescence observed in artificial tears solution after immersion of the irradiated CL for 24 h.

**Figure 5:**
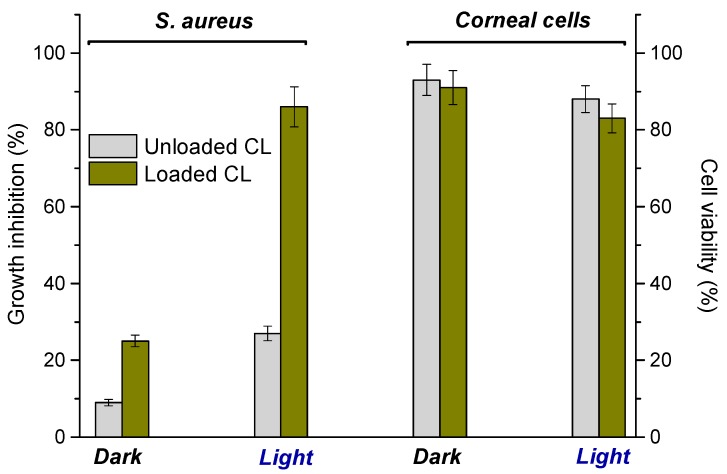
*S. aureus* growth inhibition and human corneal epithelial (HCE-2) cells viability of unloaded and loaded CL in the dark and upon illumination with visible light (λ_exc_ > 400 nm).

Antibacterial activity of CL loaded with **1** and, for comparison, unloaded CL was tested againts Gram-positive *S. aureus*, a human pathogen responsible for a significant morbidity due to the high resistance pattern towards traditional antibiotics. The bacterial cultures (*S. aureus* ATCC^®^ 6538) were incubated with the CL and kept in the dark or irradiated for 40 min with a 150 W Xenon lamp equipped with a cut-off filter at 400 nm. Parallel experiments under the same experimental conditions were also performed by incubating the CL with corneal cells. The results illustrated in Figure 5 show a negligible bactericidal activity of the CL in the dark. On the other hand, a good bacterial growth inhibition was observed for the NOPD-loaded CL under illumination reaching almost 90% inhibition, in contrast to the moderate antibacterial effects noted upon illumination in the unloaded sample. Interestingly, the control experiments carried out with all CL showed no relevant cytotoxicity on human corneal epithelial (HCE-2) cells both in the dark and under identical irradiation conditions adopted for the antibacterial test.

## 3. Materials and Methods

### 3.1. Materials

All reagents (Sigma-Aldrich, Saint Louis, MO, USA) were of high commercial grade and were used without further purification. All solvent used (from Carlo Erba, Val de Reuil, France) were spectrophotometric grade. The NOPD **1** was synthesized according to our previously published procedure [25]. The solution of artificial tears at pH 7.4 was composed of 26 mM NaHCO_3_, 18 mM KCl, 116 mM NaCl and 0.4 mM CaCl_2_. Monthly SMART-NAU CL from Safilens (Staranzano, Italy) (45% methafilcon) were used.

### 3.2. Instrumentation

UV–Vis spectra were recorded with a JascoV-560 spectrophotometer (Easton, MD, USA) using either quartz cells with a path length of 1 cm or a specific holder for thin films. Fluorescence emission spectra were recorded with Spex Fluorolog-2 (mod. F-111, Horiba Jobin-Yvon, Japan) in air-equilibrated solutions either in a right angle or in a front face mode for solution or CL samples, respectively.

Direct monitoring of NO release in solution was performed by amperometric detection (World Precision Instruments), with a ISO-NO meter, equipped with a data acquisition system, and based on direct amperometric detection of NO with short response time (<5 s) and sensitivity range 1 nM–20 µM. The analogue signal was digitalized with a four-channel recording system and transferred to a PC. The sensor was accurately calibrated by mixing standard solutions of NaNO_2_ with 0.1 M H_2_SO_4_ and 0.1 M KI according to the reaction:

4H^+^ + 2I^−^ + 2NO_2_^−^ → 2H_2_O + 2NO + I_2_

Irradiation was performed in a thermostated quartz cell (1 cm path length, 3 mL capacity) using the continuum laser with λ_exc_ = 405 nm. NO measurements were carried out under stirring with the electrode positioned outside the light path in order to avoid NO signal artifacts due to photoelectric interference on the ISO-NO electrode.

### 3.3. Chemical Detection of NO

NO release was also measured indirectly by means of the well-known, highly sensitive (detection limit on the order of the picomoles) fluorimetric bioassay of Misko et al. [30] based on the ring closure of the nonfluorescent DAN with nitrite to form the highly fluorescent product 2,3-DANT. The CL were immersed in 2 mL of artificial tears solution and exposed to daylight or kept in the dark. Afterwards, the CL were removed and 2 mL solution was added of 200 μL of DAN solution (DAN 0.30 in 0.62 M HCl) and stirred for 20 min at room temperature. Then 300 μL of NaOH 3M was added in the previous solution and stirred for 20 min at room temperature. The resultant solution was put into a quartz cell and the fluorescence emission and excitation spectra were recorded at λ_exc_ = 360 nm and λ_em_ = 405 nm, respectively. A standard calibration curve was carried out by using freshly prepared solutions of sodium nitrite in a phosphate buffer 10 mM at pH 7.4.

### 3.4. Loading of the NOPD 1 in the CL

The loading of the NOPD 1 was accomplished using the “lens soaking” technique. This technique is the simplest, most cost effective and conventional way to load drugs into CL and involves soaking the CL in the drug solution. In the present case, the CL were submerged in a NOPD solution in ethanol (0.6 mM) and stirred in the dark. The loading of the NOPD inside the CL was monitored spectrophotometrically at different times. Afterwards the CL were rinsed with water several times and left in a physiological solution. The amount of loaded NOPD was calculated considering that, at equilibrium, the hydration volume of a single CL is 16 μL (48% of polymer weight) and assuming that the molar absorption coefficient at 400 nm for 1 is 10,000 M^−1^ cm^−1^ (the same to that in ethanol solution).

### 3.5. Biological Assays

#### 3.5.1. Bacterial Killing Assay

The bactericidal activity of the CL was investigated against *S. aureus* ATCC^®^ 6538. Pure culture was inoculated in 10 mL Muller–Hinton Broth (MHB, Oxoid, Basingstoke, UK) and incubated at 37 °C for 24 h. The overnight broth culture was centrifuged at 3500 rpm for 15 min and suspended in phosphate-buffered saline (PBS). The bacterial suspension was standardized to 2 × 10^5^ colony forming unit (CFU)/mL by optical density measurements at 570 nm. One hundred µL of the bacterial suspension (20,000 CFU/mL) were placed into a 96-well microplate containing at the bottom a portion of the CL (circular disk, diameter = 6 mm) and irradiated for 40 min by using a 150 W Xe lamp equipped with a cut-off filter λ > 400 nm or kept in the dark. Afterwards, the bacterial suspension was diluted in PBS and seeded in duplicate as a spot on the Mueller–Hinton agar (Oxoid) plate. The CFUs were counted after incubation at 37 °C for 24–48 h.

#### 3.5.2. Phototoxicity Assay on Human Corneal Epithelial Cells

The photocytotoxicity experiments were carried out by irradiating for 40 min (with the same lamp used for the antibacterial test) human corneal epithelial (HCE-2) cells incubated with CL. Cell proliferation was assessed by 3-(4,5-dimethylthiazol-2-yl)-2,5-diphenyltetrazolium bromide tetrazolium (MTT) assays, based on the conversion of a substrate containing a tetrazolium ring to spectrophotometrically-detectable formazan by mitochondrial dehydrogenases. Briefly, cells were seeded at an initial density of 8 × 10^3^ cells/microwell in 96-well microplate, 200 µL of complete DMEM without phenol red was added and incubated at 37 °C for 24 h in a humidified atmosphere containing 5% CO_2_. CL were placed in the well and irradiated in conditions analogous to those described in Section 3.5.1. Thereafter, the CL were withdrawn and 20 µL of 0.5% 3-(4,5-dimethyl-thiazol-2-yl)2,5-diphenyl-tetrazolium bromide in PBS were added to each well. Following 4 h of incubation at 37 °C, the supernatant was removed and replaced with 100 µL of DMSO. The optical density (OD) was measured with a microplate spectrophotometer reader (Digital and Analog Systems, Rome, Italy) at 550 nm. Cell viability (%) was calculated from the following equation:

Cell Viability (%) = [OD_Before_ − (OD_After_/OD_Before_)] × 100

where OD_Before_ and OD_After_ are the absorbance values of the sample before and after irradiation, respectively.

## 4. Conclusions

In summary, we achieved the first example of NO photodelivering CL. This was achieved through a simple procedure which takes advantage of the effective interactions between the CL polymeric network and a tailored, water insoluble NOPD. Despite its non-covalent incorporation, the NOPD shows a high association extent to CL, which prevents any premature leaching. The photochemical properties of the NOPD are very well preserved upon its incorporation in the host CL. In fact, the loaded CL are stable in the dark towards NO release that, in contrast, is promptly observed upon daylight exposure. The sustained NO release can be followed in real-time by the intense blue fluorescence of the by-product formed concomitantly to the NO release, which acts as a convenient optical reporter of the NO concentration and provides a very easy way to check both the correct working of the doped CL and the exhaustion of the NO reservoir. Once photogenerated, the NO radical promptly diffuses out of the polymeric matrix to reach the biological target. The CL show good light-dependent bactericidal activity against the Gram-positive *S. aureus* without any relevant toxic effects on human corneal cells. These results open intriguing possibilities for further engineering of CL with NOPD in the perspective of innovative ocular devices activatable by sunlight. Experiments addressed to demonstrate the activity of NOPD-loaded CL against bacterial biofilm are currently ongoing in our laboratories.
